# Molecularly Imprinted Microrods via Mesophase Polymerization

**DOI:** 10.3390/molecules23010063

**Published:** 2017-12-28

**Authors:** Ortensia Ilaria Parisi, Luca Scrivano, Sebastiano Candamano, Mariarosa Ruffo, Anna Francesca Vattimo, Maria Vittoria Spanedda, Francesco Puoci

**Affiliations:** 1 Department of Pharmacy, Health and Nutritional Sciences, University of Calabria, 87036 Rende (CS), Italy; ortensiailaria.parisi@unical.it (O.I.P.); luca.scrivano@unical.it (L.S.); 2 Macrofarm s.r.l., c/o Department of Pharmacy, Health and Nutrition Sciences, University of Calabria, 87036 Rende (CS), Italy; mariarosaruffo8@gmail.com (M.R.); vattimoanna@yahoo.it (A.F.V.); 3 Department of Environmental and Chemical Engineering, University of Calabria, 87036 Rende (CS), Italy; sebastiano.candamano@unical.it; 4 Faculty of Pharmacy, CAMB, UMR 7199, CNRS-University of Strasbourg, 67400 Illkirch, France; spanedda@unistra.fr

**Keywords:** molecularly imprinted polymers, polymeric microrods, mesophase polymerization, theophylline

## Abstract

The aim of the present research work was the synthesis of molecularly imprinted polymers (MIPs) with a rod-like geometry via “mesophase polymerization”. The ternary lyotropic system consisting of sodium dodecyl sulfate (SDS), water, and decanol was chosen to prepare a hexagonal mesophase to direct the morphology of the synthesized imprinted polymers using theophylline, methacrylic acid, and ethylene glycol dimethacrylate as a drug model template, a functional monomer, and a crosslinker, respectively. The obtained molecularly imprinted microrods (MIMs) were assessed by performing binding experiments and in vitro release studies, and the obtained results highlighted good selective recognition abilities and sustained release properties. In conclusion, the adopted synthetic strategy involving a lyotropic mesophase system allows for the preparation of effective MIPs characterized by a rod-like morphology.

## 1. Introduction

Nowadays, particles characterized by an anisotropic shape are attracting significant interest due to their use for the production of materials with advanced microstructure, special symmetries, and unique structural, mechanical, optical, and electrical properties. Among them, rod-like polymeric particles on a micrometer scale enjoy a variety of applications as, for example, fibrillar matrices for cell attachment and growth and as delivery systems for the local release of drugs, growth factors, and bioactive molecules in tissue repair and regeneration [1].

Molecularly imprinted polymers (MIPs) have been widely used for the development of drug delivery systems (DDSs) able to release therapeutic agents in a sustainable way [2,3,4,5]. These polymeric materials are characterized by the presence of recognition cavities complementary in shape, size, and functional groups to a target molecule, which acts as a template during the polymerization process. Molecular imprinting, indeed, represents an advanced and effective technique for the preparation of polymeric matrices with selective recognition capabilities for a desired template molecule in preference to other closely related compounds and involves three main steps for the synthesis of MIPs [6]. The first one consists of the formation of a pre-polymerization complex between the template molecule and suitable functional monomers. For this purpose, the chosen monomers have to exhibit chemical structures able to interact with the functional groups of the template in a covalent or non-covalent way. The second phase involves the polymerization around the target molecule, in the presence of a porogenic solvent and a crosslinking agent, resulting in a highly cross-linked three-dimensional matrix. Finally, the template removal allows for obtaining a porous polymeric material with specific binding sites able to recognize and rebind the target molecule in a selective way.

Few examples of imprinted polymeric microrods have been reported in the literature describing the electrochemical synthesis of surface-imprinted polymers selective for protein recognition and characterized by the presence of protein-binding sites located on their surface [7,8]. The synthetic approach involves the use of sacrificial cylindrical microreactors such as track-etched polycarbonate membrane (PCM) filters. For instance nanostructured molecularly imprinted polymeric films prepared in a liquid crystalline medium (triton X-100/water) and exhibiting discernible 40 nm thick nano-fiber structures were reported by Suriyanarayanan et al. [9]. Despite the relevant progress made in the field of nanorod, nanowire, and nanotube preparation [10,11,12,13], few synthetic strategies have been established for the development of rod-like particles on a micrometer scale.

In this context, the aim of the present research study was to synthesize imprinted polymeric microrods selective for a model drug template, such as theophylline (THEO), via “mesophase polymerization”.

For this purpose, methacrylic acid (MAA) and ethylene glycol dimethacrylate (EGDMA) were used as a functional monomer and a crosslinking agent, respectively. This choice was motivated by their biocompatibility in polymerized form. Several works, indeed, report on the extensive use of MAA and EGDMA for the preparation of polymeric materials to be used in biomedical and pharmaceutical fields as drug delivery systems or for tissue engineering applications [14,15,16,17].

Polymerization within a lyotropic liquid crystalline media represents one of the most promising approaches for the synthesis of functional polymeric materials with controlled geometries and unique properties that cannot be reached using conventional bulk or precipitation polymerization.

The adoption of this kind of strategy based on mesophase polymerization for the synthesis of molecularly imprinted microrods (MIMs) represents a challenge in the world of molecular imprinting. This system could indeed interfere with the formation of specific interactions between the target molecule and the functional monomers during the pre-polymerization step, which is crucial to confer selective recognition abilities to the polymers. The strength and the stability of the initial interactions, indeed, strongly affect the selective adsorption capacities of MIPs. In the present study, the synthesis of molecularly imprinted microrods via mesophase polymerization was successfully reported for the first time.

## 2. Results and Discussion

### 2.1. Synthesis of Molecularly Imprinted Microrods (MIMs) via Mesophase Polymerization

The use of a lyotropic system as a “template” allows for the preparation of polymeric materials characterized by a highly anisotropic structure, which can be useful for a wide range of applications including ultrafiltration, biological membranes, and tissue scaffolds.

The formation of lyotropic liquid crystalline mesophases (LLCs) is due to the self-assembling of amphiphilic molecules in the presence of solvent and a number of different LLC morphologies can be achieved varying the nature of the surfactant, its concentration and temperature [18,19]. In particular, micelles are obtained at low concentrations of surfactant in polar solvent, while hexagonal and lamellar phases are observed at higher concentrations.

Three main strategies can be employed: the polymerization of conventional monomers in a non-polymerizable mesophase, the polymerization of polymerizable surfactant mesophases, and, finally, the copolymerization of traditional monomers with polymerizable surfactant mesophases [20].

In the present research work, the ternary lyotropic system consisting of sodium dodecyl sulfate (SDS), water and decanol was chosen to prepare a hexagonal mesophase, in which monomers were segregated into an ordered geometry, to direct the morphology of the synthesized theophylline imprinted polymers.

For this purpose, the three components of the lyotropic system were mixed according to a definite weight ratio, such as 29.0/66.5/4.5 (SDS/water/decanol wt %), to obtain a hexagonal mesophase characterized by rod-like micellar aggregates. Then, the previously prepared mesophase was doped with a polymerization reaction mixture, consisting of theophylline (THEO), methacrylic acid (MAA), ethylene glycol dimethacrylate (EGDMA), and ammonium persulfate, and placed at 40 °C to start the reaction, which involves a conventional radical polymerization.

It is reasonable to hypothesize that functional monomers and crosslinking molecules are segregated mainly in the hydrophilic regions and at the interface of the mesophase, respectively, adopting a geometry that directly resembles rod-like morphology. The template molecule, introduced into the system in order to synthesize the imprinted materials, interacts with the functional monomers allowing for the formation of the pre-polymerization complex, which represents the key step of the imprinting process. Moreover, the occurred template–monomer interactions work to improve theophylline solubility within the lyotropic system without affecting the doped mesophase and leading to imprinted and non-imprinted polymeric particles characterized by a similar rod-like morphology.

In order to synthesize THEO-imprinted microrods, the non-covalent approach was adopted. This methodology is based on the formation of relatively weak non-covalent interactions, such as hydrogen bonding, in both the pre-polymerization step and the rebinding phase and represents the predominant used strategy due to its flexibility, the wide range of available functional monomers and the fast kinetics of binding.

Non-molecularly imprinted microrods (NIMs) were also prepared using the same experimental procedure, but in the absence of THEO during the polymerization process, with the aim of having a control material to be used as a reference for the evaluation of imprinting efficiency and selectivity. The adopted synthetic conditions were the same for both the imprinted and non-imprinted materials, except for the presence of the template molecule during polymerization, which not only induces the formation of selective binding sites, but also affects the physical properties of such polymers. After polymerization, indeed, the template is removed obtaining a porous material that contains specific cavities able to recognize and rebind the analyte. Surface area of MIPs, therefore, tends to be greater than surface area of NIPs due to the presence of these cavities [21,22]. The NIP approach, indeed, presents one limit consisting of the different porosity of the non-imprinted material, which is usually reduced due to the absence of the template molecule during the polymerization process [23]. This reduced porosity avoids the free analyte diffusion through the NIP. On the other hand, the template removal leaves a pore channel structure within the MIP matrix, which promotes the adsorption performance of the material.

**Figure 1:**
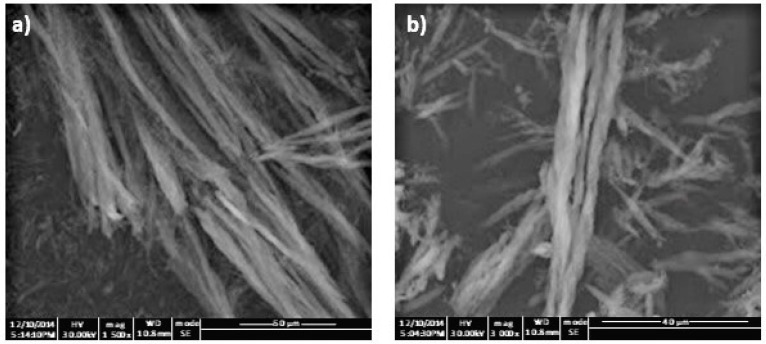
Scanning electron micrographs of the synthesized molecularly imprinted microrods (MIMs) (**a**) and non-molecularly imprinted microrods (NIMs) (**b**).

The rod-like geometry of the obtained polymers templated from the hexagonal mesophase was confirmed by SEM analysis, and no significant morphological differences between the imprinted (Figure 1a) and non-imprinted (Figure 1b) materials were observed.

### 2.2. Imprinting Effect and Selectivity Assessment

Binding studies were carried out with the aim of investigating the imprinting effect of the prepared MIMs. 

For this purpose, amounts of the imprinted and non-imprinted microrods were incubated for 3, 6, and 24 h with THEO standard solutions prepared in CH_3_CN at different concentrations, and the obtained results are reported in Table 1.

As can be seen, the best results with a difference of about 14% between imprinted and non-imprinted microrods were obtained after 24 h of incubation and using a THEO concentration equal to 0.6 mM. The higher adsorption properties of MIMs are due to the presence of binding cavities specific for the template molecule, which were formed during the polymerization process.

The selectivity of the synthesized imprinted polymers was investigated by performing the rebinding experiments using a 0.6 mM standard solution of caffeine (CAFF), which is a structural analogue of THEO. After 24 h of incubation, the amount of CAFF bound by MIMs and NIMs was practically the same and equal to 39.1 ± 0.7% and 38.3 ± 1.1%, respectively, confirming the non-specific nature of these interactions.

Molecular recognition properties and selectivity of the prepared imprinted microrods can also be expressed using two coefficients such as α and ε. The imprinting efficiency α is determined as the ratio of adsorption percentages between MIMs and NIMs for each analyte such as THEO and CAFF, while the selectivity coefficient ε is the ratio between the amount of template and analogue bound by MIMs. The α and ε values were calculated considering the binding percentages obtained after 24 h of incubation using 0.6 mM standard solutions of THEO and CAFF and reported in Table 2.

The obtained α and ε values highlighted the higher binding ability and inherent selectivity for theophylline of the imprinted microrods than the corresponding NIMs ascribable to the presence of recognizing cavities into the polymeric matrix, which are formed during the polymerization process due to the addition of template molecules. These binding cavities are characterized by a shape and an orientation of the functional groups that match the template molecules leading to specific interactions. On the contrary, the intensity of the formed interactions is very weak in the absence of recognizing sites.

### 2.3. In Vitro Drug Release Studies

The ability of MIMs and NIMs to act as controlled drug delivery systems was assessed in phosphate buffered saline (PBS) at pH 7.4 by the dialysis bag diffusion technique.

After the impregnation step, the drug loading content (DLC) and the drug loading efficiency (DLE) were calculated based on Equations (1) and (2), respectively:

DLC (%) = W_ld_/(W_ld_ + W_mr_) × 100 (1)

DLE (%) = W_ld_/W_d_ × 100 (2)

in which W_ld_, W_d_, and W_mr_ are the weight of the loaded drug within the microrods, the weight of the total drug used for the impregnation procedure, and the weight of the dried microrods, respectively. The obtained DLC and DLE values are reported in Table 3.

The imprinted microrods loaded a higher amount of THEO during the impregnation procedure than the corresponding non-imprinted rod-like particles due to their greater affinity for the drug molecule. During the polymerization process, indeed, the presence of the template molecule allows for the formation of recognition sites inside the polymeric matrix, which are responsible for the higher tendency of the imprinted material to adsorb theophylline.

**Figure 2:**
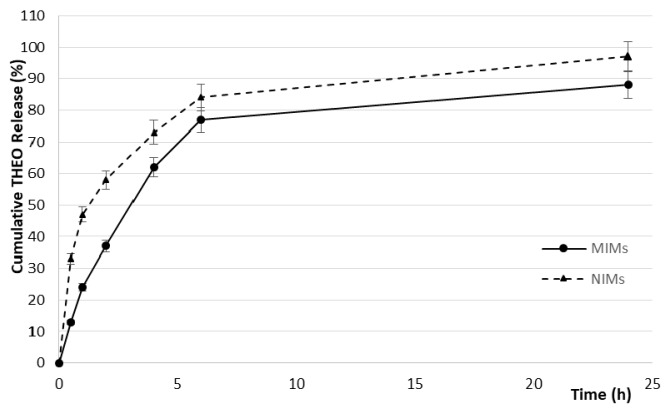
THEO release profiles.

Furthermore, the presence of binding cavities results in a controlled THEO release by MIMs, which is more evident during the first stages (Figure 2).

This typical behavior of the imprinted polymers prevents the so-called burst release and, therefore, the rapid diffusion of the therapeutic agent. During the first two hours, indeed, 58% of the loaded THEO was released by the non-imprinted microrods; on the other hand, only 37% of the drug was released by MIMs.

## 3. Materials and Methods

### 3.1. Materials

Sodium dodecyl sulfate (SDS), theophylline (THEO), caffeine (CAFF), methacrylic acid (MAA), ethylene glycol dimethacrylate (EGDMA), ammonium persulfate, disodium hydrogen phosphate, sodium dihydrogen phosphate were purchased from Sigma-Aldrich (Milan, Italy).

In the aim to remove stabilizer and impurities, methacrylic acid was purified before use by a single-step passage through an alumina column.

All solvents were reagent or HPLC grade and supplied by VWR (Milan, Italy).

Dialysis membranes of 6–27/32” Medicell International LTD (MWCO: 12–14,000 Da) were employed for the in vitro release studies.

### 3.2. Instrumentation

The HPLC analyses were carried out using a Jasco PU-2080 liquid chromatograph (Tokyo, Japan) equipped with a Rheodyne 7725i injector (fitted with a 20-µL loop), a Jasco UV-2075 HPLC detector and a Jasco-Borwin integrator (Jasco Europe s.r.l., Cremella (LC), Italy). The adopted HPLC conditions for theophylline and caffeine analysis were previously reported in literature [24].

The scanning electron microscopy (SEM) micrographs were obtained with a Jeol JSMT 300 A (JEOL (ITALIA) S.p.A., Milan, Italy); the samples were made conductive by gold layer deposition on sample surfaces in a vacuum chamber. The particle size distribution range was calculated employing an image processing and analysis system, a Leica DMRB endowed with a LEICA Wild 3D.

### 3.3. Synthesis of Molecularly Imprinted Microrods (MIMs) via Mesophase Polymerization

The developed synthetic procedure involves two main consecutive steps: the preparation of the hexagonal mesophase and the radical polymerization.

The hexagonal mesophase based on the ternary lyotropic system consisting of SDS/water/decanol (29.0/66.5/4.5 wt %) was prepared as reported in the literature with suitable modifications [25]. In a sealed flask, 2.9 g of SDS were dissolved in 6.65 mL of water and, decanol was then added to the solution. The obtained mixture was homogenized by vortex mixing and mechanical stirring, and the flask was then kept at 35 °C under magnetic stirring. After 24 h, the obtained mesophase was doped with template, functional monomer, crosslinker, and initiator for the polymerization step using a THEO/MAA/EGDMA molar ratio equal to 1:8:20. For this purpose, 180 mg of theophylline and 0.68 mL of MAA were dissolved in 3 mL of CHCl_3_ and the obtained mixture was sonicated for 10 min in order to promote the formation of the pre-polymerization complex. Finally, 3.77 mL of EGDMA and 100 mg of ammonium persulfate were added to the reaction mixture, which was purged with nitrogen and sonicated for another 10 min. The polymerization mixture was added to the previously prepared mesophase, homogenized by mechanical stirring and, finally, polymerized by keeping the system at 40 °C for three days.

The obtained polymeric materials were washed with water in order to remove the surfactant, ethanol, acetone, and diethyl ether by centrifugation at 10,000 rpm for 10 min. A further washing step for template removal was carried out by Soxhlet extraction using 200 mL of an acetic acid/methanol (1:9, *v*/*v*) mixture for at least 4 h, followed by 200 mL of methanol for another 4 h. Finally, the polymeric particles were dried under vacuum overnight at 40 °C. 

MIMs were checked to be free of THEO and any other compound by HPLC analysis.

Non-molecularly imprinted microrods (NIMs) were also synthesized using the same experimental procedure, but in the absence of THEO during the polymerization process, and treated in the same conditions.

### 3.4. Binding Studies: Imprinting Effect and Selectivity Assessment

With the aim of investigating the selective recognition properties of the synthesized microrods, binding studies were carried out as described below.

Fifty milligrams of polymeric imprinted and non-imprinted microrods were mixed with 0.5 mL of CH_3_CN and 0.5 mL of a THEO standard solution in CH_3_CN. Samples were shaken in a water bath at 37 ± 0.5 °C for 3, 6, and 24 h and centrifuged at 12,000 rpm for 20 min, and the concentration of free THEO in the supernatant was finally measured by HPLC analysis. 

The binding experiments were carried out using different THEO standard solutions in order to study the effect of theophylline concentration.

MIM selectivity was evaluated by performing binding studies under the same experimental conditions but using a standard solution of caffeine, which is a THEO analogue.

All experiments were repeated three times.

### 3.5. Drug Loading by Soaking Procedure and In Vitro Release Studies

Theophylline loading was achieved by immersing 180 mg of imprinted and non-imprinted microrods in 3 mL of a THEO solution in CH_3_OH (37 mM). The obtained suspensions were soaked in dark conditions and, after 24 h, were placed in a sintered glass filter in order to remove the excess of solvent by percolation at atmospheric pressure. Then, the resultant polymeric microrods were dried under vacuum overnight, and the amount of non-loaded THEO in the excess of solvent was measured by HPLC analyses. Finally, the quantity of loaded drug by imprinted and non-imprinted matrices was calculated by difference from the total amount.

The dialysis bag diffusion technique was employed in the in vitro release studies. For this purpose, 10 mg of loaded MIMs and NIMs were introduced into dialysis membrane bags, which were immersed in 10 mL of PBS (0.01 M) at pH 7.4 and maintained at 37 ± 0.5 °C in a water bath with horizontal shaking. After pre-determined time intervals (0.5, 1, 2, 4, 6 and 24 h), 3 mL of the dissolution medium were withdrawn and replaced with the same volume of PBS. The amount of released theophylline was quantified by HPLC analysis, and the percentage of released drug was calculated considering 100% of the THEO content in polymeric microrods after the drying procedure. The cumulative amount was plotted as a function of time.

Experiments were repeated in triplicate.

## 4. Conclusions

In the present research study, the synthesis of MIMs selective for theophylline via mesophase polymerization was successfully reported for the first time using methacrylic acid and ethylene glycol dimethacrylate as functional monomer and crosslinker, respectively. The choice of this polymer composition is due to the confirmed biocompatibility of polymeric materials based on MAA and EGDMA and widely employed in biomedical and pharmaceutical fields.

For this purpose, the ternary lyotropic system consisting of SDS, water, and decanol was chosen to prepare a hexagonal mesophase, which acts as a “template” in order to impart a rod-like morphology to the synthesized polymeric particles. The obtained MIMs have been shown to have good selective recognition abilities and controlled release properties. Furthermore, during the impregnation process, the imprinted microrods were able to load a higher amount of THEO compared to the corresponding non-imprinted materials.

The observed properties depend on the affinity of the imprinted matrices for the target molecule due to the presence of specific recognition cavities, which are formed during the polymerization.

Therefore, based on the obtained results, it is possible to state that the adopted synthetic strategy involving a lyotropic mesophase system does not negatively interfere with the formation of the pre-polymerization complex or thus with the imprinting properties of the prepared polymers. 

Future studies will involve the use of bioactive molecules as a template for the preparation of MIMs via mesophase polymerization, which has potential for use as active tissue scaffolds able to release templates in a controlled manner during tissue repair and regeneration processes.

## Figures and Tables

**Table 1:** Percentages of bound theophylline (THEO) by imprinted (MIMs) and non-imprinted (NIMs) microrods. Data are shown as means ± S.D.

Incubation Time (h)	Bound THEO (%) 0.6 mM		Bound THEO (%) 0.06 mM	
	MIMs	NIMs	MIMs	NIMs
3	45.8 ± 1.1	58.4 ± 0.8	23.4 ± 1.0	33.2 ± 1.1
6	50.6 ± 0.9	42.5 ± 0.7	42.7 ± 0.6	31.5 ± 0.7
24	60.7 ± 0.8	46.6 ± 0.7	49.2 ± 0.7	39.1 ± 1.2

**Table 2:** Imprinting efficiency (α) and selectivity coefficient (ε).

Imprinting Efficiency (α)		Selectivity Coefficient (ε)
α_THEO_	α_CAFF_	
1.3	1.0	1.5

**Table 3:** Drug loading content (DLC) and drug loading efficiency (DLE).

DLC (%)		DLE (%)	
*MIMs*	*NIMs*	*MIMs*	*NIMs*
8.9 ± 0.6	7.7 ± 0.8	89.0 ± 0.7	76.5 ± 0.8
